# Attention and Information Acquisition: Comparison of Mouse-Click with Eye-Movement Attention Tracking

**DOI:** 10.16910/jemr.11.6.4

**Published:** 2018-11-16

**Authors:** Steffen Egner, Stefanie Reimann, Rainer Hoeger, Wolfgang H. Zangemeister

**Affiliations:** MediaAnalyzer GmbH, Hamburg, Germany; University of Luneburg, Germany; University of Hamburg, Germany

**Keywords:** Visual Attention, Information acquisition, Mouse-Click Attention Tracking, Eye-Movement Attention Tracking, Comparison of Attention Tracking, Visual search, Scanpath

## Abstract

Attention is crucial as a fundamental prerequisite for perception. The measurement of attention in viewing and recognizing the images that surround us constitutes an important part of eye movement research, particularly in advertising-effectiveness research. Recording eye and gaze (i.e. eye and head) movements is considered the standard procedure for measuring attention. However, alternative measurement methods have been developed in recent years, one of which is mouse-click attention tracking (mcAT) by means of an on-line based procedure that measures gaze motion via a mouse-click (i.e. a hand and finger positioning maneuver) on a computer screen.

Here we compared the validity of mcAT with eye movement attention tracking (emAT). We recorded data in a between subject design via emAT and mcAT and analyzed and compared 20 subjects for correlations. The test stimuli consisted of 64 images that were assigned to eight categories. Our main results demonstrated a highly significant correlation (p < 0.001) between mcAT and emAT data. We also found significant differences in correlations between different image categories. For simply structured pictures of humans or animals in particular, mcAT provided highly valid and more consistent results compared to emAT. We concluded that mcAT is a suitable method for measuring the attention we give to the images that surround us, such as photographs, graphics, art or digital and print advertisements.

## Introduction

Visual Attention is a research topic of increasing impact. Not only is attention an interesting topic in itself, it also plays a crucial role in perception and motor control. Moreover, measuring attention also yields valuable data for studying higher cognitive functions such as interest, understanding and reading. Measurement of attention is traditionally being seen as parallel to eye-tracking (1, 2 ,3 ,4 ,5) the underlying rationale is that humans direct the region of their retina with the highest resolution (fovea) to aspects of the optical scene which are of high relevance for the organism. However, experiments with response latency tasks (7) clearly indicate that there are attention shifts that are not measurable as eye movements (covert attention). Moreover, the visual modalities do not seem to be specifically linked to attention. In fact, attention seems to be modality-unspecific (8, 9). The two classical ways to measure attention have specific advantages and disadvantages.

### Eye-Tracking and attention

As indicated by its name, eye tracking measures the position and orientation of the eye(s). Based on these raw data, gaze position in the environment can be determined. Eye-tracking is a technique to measure an individual's visual attention, focus, and eye movements. This experimental methodology has proven useful both for human-computer interaction research and for studying the cognitive processes involved in visual information processing, including which visual elements people look at first and spend the most time on (10).

Fixation criteria are often unclear. Blinks, correction saccades, physiological and technical noise contribute to difficulty of measurement: This is attention tracking by eye movement – emAT.

Response latency tasks assume that the reaction upon an event that happens at a specific location – for instance the onset of a stimulus – will be quicker when the position of the emerging stimulus is expected at that particular moment. This method allows the measurement of covert attention (7), which precedes eye-movements in some cases. Each trial of a respondent only reveals one attended location at the most. Therefore, we cannot measure a full path of attentional shifts, but only individual locations. Some authors propose that attention can be measured in other ways than the two methods mentioned above.

Some of these other ways employ the computer mouse to indicate attention locations. One of these methods, mouse-click based attention tracking (mcAT) will be examined in more detail in this paper. It seems obvious that a method that relies on the computer and a mouse as the only necessary devices would have many practical advantages over other methods. But the question is: Is it a method that generates results with validity comparable to eye tracking?

Both, *salience and conspicuousness* of a stimulus in terms of its environment (11) as well as *relevance of a stimulus* are decisive criteria for the allocation of attention. They seem to be based upon two independent systems (12); however, to quantify the impact of bottom-up and top-down mechanisms within a certain setup may be highly difficult to differentiate with respect to their individual importance to the actual perception (13, 14). Various studies have shown that the degree of exogenous and endogenous direction of attention depends upon a number of factors. Novel or unfamiliar stimuli situations in free-viewing tasks, or the viewing of images in advertising are usually thought to be dominated by bottom-up processes, especially at the beginning of the viewing time (15, 16, 17). However, with increased viewing time, and with known visual performances and situations as well as in the search for certain stimuli, the situation is dominated by top-down processes.

Viewing time: The total amount of time within an AOI approximately complies with the fixation duration – the time between two successive clicks, generally half the fixation before (max. 500ms) and half of the fixation attributed thereafter (max. 500 ms).

### Selective attention and eye movements – the classical relationship to study.

Selective attention is the gateway to conscious experience, affecting our ability to perceive, distinguish and remember the various stimuli that come our way (18). Selective attention denotes the allocation of limited processing resources to some stimuli or tasks at the expense of others (19, 20, 21, 23, 24). Apart from its effects on perception or memory, selective attention is a significant contributor to motor control, determining which of the various objects in the visual field is to be the target used to plan and guide movement. As selective visual attention allows us to concentrate on one aspect of the visual field while ignoring other things, it is modulated by both involuntarily bottom-up and voluntary top-down mechanisms (20), within a brainstem-parietotemporal and basal ganglia-frontal neuronal network (25).

Selective visual attention for spatial locations is under the control of the same neural circuits as those in charge of motor programming of saccades (26, 27, 28, 29, 30).

Directing visual attention to a certain location as well as ocular saccades in visual attention tasks depend upon accurate saccade programming. Programming the eye saccade is thought to lead to an obligatory shift of attention to the saccade target before the voluntary eye movement is executed, which is due to two parameters: correct programming of the saccade and correct saccade dynamics. (31, 32, 33, 34).

Therefore, the alertness of central, top-down programming influences oculomotor function and, conversely, a resulting oculomotor dysfunction could have a direct, bottom up impact on results of visual attention tasks.

Visual selective attention can be investigated by visual search tasks.

Visual search means to look for something in a cluttered visual environment. The item that the observer is searching for is termed the target, while non-target items are termed distractors. Many visual scenes contain more information than we can fully process all at once. Accordingly, mechanisms like those subserving object recognition might process only a selected/restricted part of the visual scene at any one time. Visual attention is used to control the selection of the subset of the scene, and most visual searches consist of a *series of attentional deployments*, which ends either when the target is found, or when the search is abandoned. Overt search refers to a series of eye movements around the scene made to bring difficult-to-resolve items onto the fovea. Only if the relevant items in the visual scene are large enough to be identified without fixation can the search be successfully performed while the eyes are focused upon a single point. In this case, attentional shifts made during a single fixation are termed *covert*, because they are *inferred rather than directly observed*.

While under laboratory conditions, many search tasks can be performed entirely with covert attention, under real world conditions a new point of fixation is selected 3 to 4 times per second. *Overt fast movements of the eye, saccades, and covert deployments of attention are closely related* (20), as the sample rate of saccades is 4/sec. With stimuli that do not require direct foveation, 4–8 objects can be searched during each fixation. As estimates of the minimum time required to recognize a single object are almost always greater than 100 ms, multiple items may be processed in parallel (35). *Volitional* deployments of attention are much slower than *automatic* deployments (36), and occur at a rate similar to saccadic eye movements, i.e. a sample rate of 4/sec (37). *Search termination* happens after finding the target, or one could declare the target to be absent after rejection of every distractor object, although it may be difficult to determine when this point has been reached.

### Mouse-click attention tracking – Background

The mouse-click Attention Tracking (mcAT) method measures attention by mouse clicks. They can be counted and concatenated to a time sequence that is analogous to the eye movement scanpath. Egner and Scheier developed this method in collaboration with Laurent Itti (38) at the California Institute of Technology (USA). They assumed the predictive power of a computerized attention model with three categories of visual stimuli (photographs of natural scenes, artificial laboratory stimuli and sites).

Based upon empirical evidence of a close link between attention, eye movements/fixations and pointing movements (39) the eye tracking data (emAT), touch screen and click data with a computer mouse (mcAT) were highly correlated (an overview can be found in (16, 38, 40). The mcAT method was patented in the US and Europe as a mouse-click based AT procedure for measuring visual attention (41, 42).

The central idea of the mcAT method is the natural coupling of the use of mouse clicks with eye movement measures, which in turn represent a valid indicator of the attention.

In the following years, also other researchers have explored the relationship between users' mouse movements and eye movements on web pages (43).

Deng, Krause and Fei-Fei (44) used a bubble paradigm of Gosselin and Schyns (45) that was used to discover the object/image regions people explicitly choose to use when performing.

Chen, Anderson and Sohn (46) described in their paper a study on the relationship between gaze position and cursor position on a computer screen during web browsing. Users were asked to browse several web sites while their eye/mouse movements were recorded. The data suggested that there was a strong relationship between gaze position and cursor position. The data also showed that there were regular patterns of eye/mouse movements. Based on these findings, they argued that a mouse could provide more information than just the x, y position where a user was pointing. They speculated that by understanding the intent of every mouse movement, one should be able to achieve a better interface for human computer interaction.

Using eye and mouse data, Navalpakkam et al. (47) demonstrated that the mouse, like the eye, is sensitive to two key attributes of page elements: their position (layout), and their relevance to the user's task. They identified mouse measures that were strongly correlated with eye movements and developed models to predict user attention (eye gaze) from mouse activity.

Our approach is different from the viewing window approach of (44) in that we explicitly collect the path of discretized click data, as each click represents a conscious choice made by the user to reveal a portion of the image. Since the clicks correspond to individual locations of attention, we can directly compare them to eye fixations.

Kim et al. (48) investigated the utility of using mouse clicks as an alternative for eye fixations in the context of understanding data visualizations. They developed a crowdsourced study online in which participants were presented with a series of images containing graphs and diagrams and asked to describe them. They compared the mouse click data with the fixation data from a complementary eye-tracking experiment by calculating the similarity between resulting heatmaps and got a high similarity score and suggested that this methodology could also be used to complement eye-tracking studies with an additional behavioral measurement, since it is specifically designed to measure which information people consciously choose to examine for understanding visualizations.

### Aim of our study

The question is, can mouse clicks approximate human fixations in the context of data visualization understanding? When we compare eye movement/fixations and hand mouse movement/clicks, we assume that the sensory-attentional and the cognitive part of these actions are highly similar, whereas the motor part is obviously different. From this reasoning we can infer three questions:

1. What *are* the differences between eye and hand movements that have been described by many researchers (49, 50) and how do they relate to our findings?
2. What are the similarities of the two responses?
3. Are there non-motor differences related to attention/cognition and how do they relate to our findings?

The present study is of interest to the eye movement research community for the following reasons. While eye-tracking is the well-established method for measuring visual attention, the eye movement data does not allow to make a distinction between eye-movement-specific and attention-specific effects. The alternative measurement described and used in the present article uses the hand (computer mouse) to measure attention. The resulting data is highly similar to eye-tracking data, and it is not affected by eye-movement-specific processes. Thus, it allows to separate eye-movement-specific and attention-specific effects. Additionally, the alternative measurement enables a method comparison, which enriches our knowledge about eye-tracking methodology. Last, the new method can help to gain a better understanding of attention, which is also the goal of much eye and mouse tracking research on web page viewing. So, both methods contribute to the same goal.

Therefore, two practical issues were addressed:

- Does the spatial dimension of fixations and clicks correlate highly positively?
- Does this putative correlation depend upon the stimulus material?

In view of our experiences, we hypothesized that:

- The overall pattern of fixations/clicks correlate highly.
- The amount of the agreement between both recording methods depends upon the nature of the stimuli.

## Methods

We used an independent experimental design i.e. a between-subject design selected to prevent both carry-over effects (affecting a later experimental condition by a previous condition) as well as to prevent position effects like fatigue and exercise.

### Subjects 

The data of twenty participants were used. One group of subjects underwent one experimental condition only: In ten subjects emAT was measured via eye movement recordings while viewing the stimulus material.

The remaining ten subjects were subject to mcAT, measured via mouse-click recordings while viewing the stimulus material. All other experimental conditions (stimulus material, type of presentation, site of examination, demographics of the subjects, experimenter) were kept constant. Before the actual test took place, multiple testing of subjects to clarify, check and optimize instructions and operation of the equipment took place.

The study has been approved by the local ethics committee. It complies with the ethical practices and follows the Code of Conduct and Best Practice Guidelines outlined by the Committee on Publication Ethics. Informed consent for the research was obtained by an oral introduction and overview at the LEUPHANA University of Luneburg, Engineering Psychology research lab.

### Sample

The whole study consisted of three phases: (1) compilation of the stimulus material, (2) pilot run, and (3) final experiment.

The participants of all three trials – of the preliminary selection of images and classification in the classification scheme (n = 12), the trial run (n = 6) and the final test with emAT and mcAT measurements (n = 29) – were students recruited from the University of Lueneburg. All participants gave their written informed consent following the rules of the Helsinki Declaration. None of the subjects (Ss) had participated in more than one of the tests or knew about the exact purpose of the investigation.

The comparison of various studies shows a wide range in the number of required study participants. An overview of Borji and Itti (11) lists over 19 trials in which attention stimuli computer programs were used based on emAT; the subject numbers vary between 5 and 40, but more than half of the listed studies used less than 15 subjects. To keep the failure rate of the emAT test low, only subjects that did not rely on visual aids were invited.

Four out of 14 emAT respondents were removed from the evaluation. The criterion for removing such recordings was the calibration quality. The calibration, which was performed in the beginning of the recording, was checked at the end of each recording. The respondent had to redo the calibration procedure. If the results were significantly different from the initial calibration, we removed the recording. This was decided upon face validity. The reasoning behind this is that, if the calibration parameters have changed throughout the recording process, this is due to a distortion that happened during the recording. The remaining ten were between 18 and 22 years old (average age: 20). Four were female. Also, five out of 15 mcAT respondents were removed from the evaluation. This was decided upon the mouse behavior during the recording. The recording was excluded if:

- the click rate went below 1.5 clicks per second,
- the respondent stopped moving the mouse,
- the click pattern revealed that the respondent did not understand the instructions. The last point was decided upon face validity.

Subjects for mcAT measurements had no restrictions concerning visual aids. Of the 15 published subjects, ten recordings could be used for further analysis. From the ten remaining subjects, the age range was 20–26 years (average age: 23) and the sex ratio was even.

### Stimuli

Until now, no generally accepted classification schemes, neither number nor type of classes have been suggested in attention research. Examples of the classification of the photos are: Natural landscapes and portraits (51); animals in natural environments, street scenes, buildings, flowers and natural landscapes (52); nature/landscape scenes, urban environments and artificial laboratory stimuli (53) as well as images with obvious and unclear/non-existent AOI (54). None of the authors stated the reasons/justification for the particular classification that was selected. Therefore, we developed our own classification scheme that reflects the suggestions in the literature but has also been derived from features that mirror the mechanisms of control of attention.

**Figure 1.**
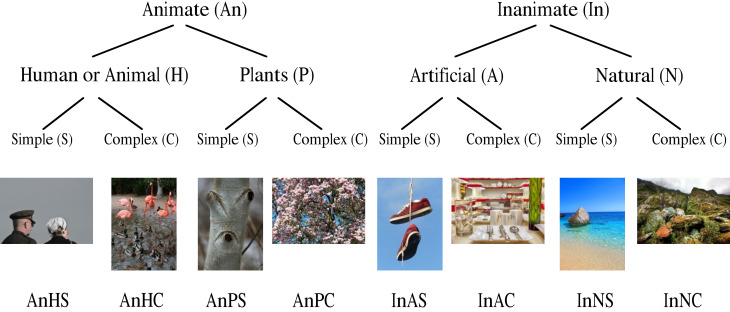
Images and their category levels. Utilized stimulus material: human/animal, easy, complex, plants, inanimate, artificial, natural

Figure 1 shows the distribution of images at their different levels. At the highest level were photographs that represented animate and inanimate matter. At the second level, the class animate represented pictures of people/animals-plants; the inanimate class included artificial/natural environments. Each of these four classes is independent of content aspects and divided into simple and complex designs.

Although complexity in different contexts may be an important determinant of attention processes (12, 55), different authors define the term complexity in many different ways.

### Image selection

The photographs originate from two image databases: Borji et al. (56) which is currently the largest, freely available and commonly used image data set that was also used by Judd et al. (51). Another source was photos from the pixabay.com website, a database for a Creative Commons CCO.

First, we chose a preselection of images per category (120). To ensure that the classification of images was done objectively despite different possible interpretations of schema classes, we reduced the dataset to eight photos per category using 12 subjects for classification. The subjects were given the task of classifying each of the 120 sequentially presented images in the classification scheme shown above without further explanation of the schema classes. In this way, an impartial classification could be performed.

Only those images were chosen for the experiment for which a minimum of two thirds of the subjects chose a particular category.

During the evaluation, it was found that some subjects had difficulties differentiating between the categories “natural” and “plants”; the terms “complex” and “simple” were also interpreted quite differently. Subsequently, the selected 64 images were cut to three uniform sizes to reflect the screen used in the following test: 8 photos are dimensioned in portrait format (690 x 920 pixels); 30 photos are in landscape format (1226 x 920 pixels); 26 photos are in landscape format with 1250 x 833 pixels.

### Distribution of image material at different levels. 

Photographs with representation of animate and inanimate matter were at the highest level. The second level, the “animate” class, represented images of people/animals, and plants, and the “inanimate” class in artificial and natural environments. Each of these four classes was again independent of content aspects and divided into simple and complex designs. Although complexity was in different contexts an important determinant of attention processes (12, 55) many authors have used the term differently.

### A priori AOIs – grid application

To compare the spatial distribution of the viewing and click data in a meaningful way, regions (ROI) or areas of interest (AOI) had to be defined. For this, semantically-based AOIs have been frequently used, in particular in the analyses of advertisements or sites. As we were less interested in gaze behavior with respect to specific image regions and were more interested in the global eye movements over the entire image, we used a grid laid over the image that divided the image into a certain number of fields. In this way, we excluded subjective preferences that might confound the results of our analysis. Of course, this procedure had its disadvantages: The choice of the grid field’s size sometimes played a particularly important role by defining objects too inaccurately, e.g. a face might be divided into several fields and, therefore, subdivided by single fixations. As the image data set used here contained many complex stimuli for which AOIs were difficult to define, gridding was the only meaningful way to analyze the data. As a compromise, we selected a 5 x 7 grid (35 fields), so that the fields had an average square size. The attention parameters were steadily distributed features and could assume values between 0.0 and 1.0. For example, a value of 0.24 in a grid meant that 24% of all clicks or fixations were made in this particular field. The contact parameters, however, were discretely distributed. 24% of all clicks or fixations were made in this field. The contact parameters, however, were discretely distributed features, with eleven possible specifications between 0 and 1 (in increments of 10) for ten subjects; for example, a value of 0.3 means that 30% of the subjects had looked or clicked in a field.

An arbitrarily chosen grid definitely has disadvantages in the evaluation. Among other things, adjacent fixations (or clicks) may fall into separate grid cells, even though both fixations belong to the same object. It would be desirable to evaluate fixations on the same object together. As an alternative to the grid approach, one can also define regions. Ideally, the regions are set to correspond to fixation goals (objects). This bypasses the above cited disadvantage. However, this approach also has a significant disadvantage. The manually selected regions can strongly distort the results if chosen unfavourably. They could also be used to deliberately distort results. We will not solve this general problem of the eye tracking community with our article (see (57) for a methodological overview). That is beyond the scope of our paper. The JEMR paper by Oliver Hein and Wolfgang H. Zangemeister (58) offers one possible solution.

In summary, to calculate the fixation and click data, the *contact value* was calculated, i.e. the proportion of subjects that viewed or clicked in a particular field. Also, the *attention value* of the subjects was calculated by averaging the *single grid* percentage clicks or fixations with respect to the total clicks or fixations per image.

For automatic algorithmic generation of particular grid sizes and/or content specific AOIs see: Privitera and Stark (59) and Hein and Zangemeister (58).

### Experimental Setup

In order to keep the experimental conditions for both measuring methods as constant as possible, data collection was carried out in both emAT and mcAT in the eye movement laboratory of the University of Lüneburg between November 1 and 16, 2015. The stimulus material was presented on a 21.5-inch monitor (Acer) with a resolution of 1920 x 1080. To avoid sequence effects, the 64 images in both experiments were presented in randomized order each for the duration of 5 s. This timing was chosen in accordance with many other related studies (52, 54, 60). We separated the individual images by means of a blank screen (here for a duration of 2 s) on which a commonly used fixation cross was shown in the middle (61). A fixation cross was used for both measurement methods for all pictures to ensure a common starting position for the eyes and the computer mouse. Following both tests, the subjects answered a short questionnaire on their demographic data. They also completed a recognition test and had to judge whether a series of images in the previous experiment was shown or not. The mcAT-subjects were also requested to answer three qualitative questions with click behavior.

### Eye movement Attention Tracking (emAT)

The eye movement measurement was carried out with the SMI iView X™ Hi-Speed 1250 eye tracker. This is a tower-mounted dark-pupil system recording movements of one eye with a sampling rate of 500 Hz (SMI SensoMotoric Instruments GmbH; SMI SensoMotoric Instruments GmbH). The distance between the chin rest of the iView X™ and the screen was 60cm. Programming, evaluation and control of the experiment was carried out using SMI’s BeGaze Analysis (version 3.5). The iViewX program was used to control the recording of eye movement.

Subjects received standardized verbal instructions, during which they were informed of the calibration and test procedure. Automatic calibration then followed, with a spatial accuracy of at least 0.5°. We used additional manual calibration in case accuracy was insufficient. This took place before and after the presentation of 64 images for 10 seconds for each image. Thus, both the measurement accuracy and precision were validated to provide assessment of data quality. After the first calibration, further instructions were carried out on the screen. Subjects were asked to view the following images as they chose in a free-viewing task to create almost natural viewing conditions without any viewing strategies (see as refs.: Borji & Itti, (11); Parkhurst et al. (14)). Overall, the presentation of images took about ten minutes.

### Mouse click Attention Tracking (mcAT)

For the mcAT test, the instructions (Appendix 1), click training, click test with 64 images and the demographic data collection was programmed using MALight software from MediaAnalyzer in an online questionnaire. While the subjects initially completed the click training, the experimenter looked to answer questions and provide guidance for clicking behavior in case the instructions were not understood. The subsequent viewing of images was done similarly to the emAT without any task, with the supplementary advice: “You can click everywhere you are looking at”. Completion of the click training, and the click test took about 12 minutes.

### Data Analysis

Default settings algorithm parameters of “BeGaze” (SMI SensoMotoric Instruments GmbH) were: Saccade detection parameters: min. duration 22ms, peak velocity threshold 40°/s, min. fixation duration 50ms. Peak velocity start: 20% of saccade length; end: 80% of saccade length.

First the emAT fixations were calculated. BeGaze contained both a dispersion-based and a speed-based algorithm, which was used here because the SMI machine is a high-speed device. The minimum fixation duration was based upon inspection of selected images and subjects for durations of 50 ms, 100 ms and 200 ms. Based upon this visual analysis and the information in the literature of Holmqvist et al. (61), we used a minimum fixation duration of 100 ms (instead of the default 50 ms) as the parameter setting.

### Data Cleansing

Next, the data quality of each subject was checked by their fixations at the beginning and at the end of the experiment. In case deviation between initial and final calibration fixations (precision and accuracy) was too high, we had to exclude four subjects (s. Annex 4). It can be assumed that the first specific fixation does not necessarily start with the very first fixation, but after a certain period of time that we defined to be 500ms: Therefore, the first 500ms were excluded from further evaluation. Compared to the first saccadic eye fixations, the manual start of the mouse clicks i.e. the “mouse-fixation” was slightly slower than the sequence of eye movement fixations, due to the inertial load difference between eye and hand. Therefore, for mouse clicks we excluded the first 800ms from further evaluation. Decisive for the quality of click data was a minimum click speed that can be controlled. The demanded click rate was 1.5 per second or higher. This was attained by all subjects. The click data in the attention and contact parameters were transformed per grid and averaged across all subjects by means of Microsoft Excel. At 35 fields per frame and a total of 64 images, 2240 values per sample were observed for each method (mcAT and emAT).

Click test. Subjects view a series of stimuli on a screen for 5–7 seconds – usually advertising materials, website or shelf view, mostly in combination with distractors that are shown before and after the test material. They are prompted to click quickly and without thinking on those places that they consider to be attractive. This measurement is carried out as a “click test” during an online survey, which they can have performed by an online panel of recruited volunteers with their own computers at home. As the subjects are required to perceive the mouse as an “extension” of the eye, a short time of training for the exercise is required. This click training is an interactive and playful method, based upon five tasks during which the subjects get accustomed to clicking continuously fast – at least 1–2 times per second – while they control certain image regions with the mouse. Meanwhile, they receive real-time evaluation feedback on their click behavior. Only subjects that pass all tasks, i.e. also after several attempts have been successfully completed, can they take part in the next click test. The aim of the training is to teach subjects to click as spontaneously and unconsciously as they direct their gaze, so that a fixation and a mouse-click become equivalent.

The data collected from the click test and the survey are stored on a server, and MediaAnalyzer uses special software to statistically analyze and interpret the data. Firstly, there is a verification of data quality and possibly a data cleansing. Despite click training, few subjects fail to maintain the clicks throughout the test or show a lack of motivation by clicking only on the same spot. Data from these subjects can be detected and filtered by algorithms (39, 40, 62). During the evaluation, a click is taken as a fixation. It is analyzed similarly to an etAT based upon semantically-derived, predefined areas of interest (AOIs) – at an ad e.g. based on the logo or the name – and on the average results of all subjects.

Typical parameters are:

i. Time to contact: The time to first click in an AOI

ii. Percent attention: Share of clicks in an AOI relative to the total number of click-stimulus corresponds approximately to the relative fixation frequency; thereafter referred to as attention value.

iii. Percent contact: Relative proportion of subjects that clicked at least once in an AOI; thereafter referred to as contact value.

### Statistical analysis

Using IBM SPSS, various summary measures were calculated to determine the relationship between the converted data from mcAT and emAT: Pearson product-moment correlation coefficient, the area under curve (AUC) and the receiver operating characteristic (ROC) curve.

The correlation analysis and the calculation of the ROC curve are the most commonly used methods for analyses of these data (53, 56, 63). The use of two or more evaluation methods is recommended to ensure that the observed effects are independent of the summary measure (56).

**Figure 2.**
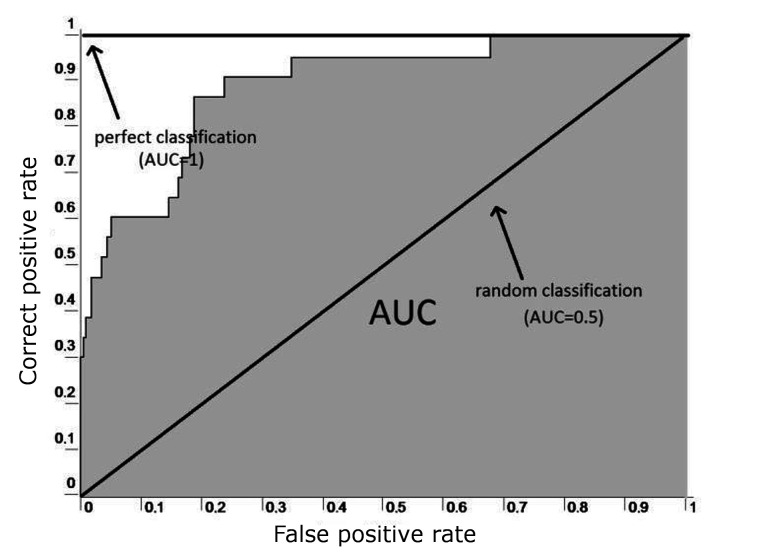
ROC curve example (5, 6).

The *Pearson product-moment correlation coefficient* provides information about the amount and direction of the linear relationship between two interval-scaled variables, in this case between the pairs of values of the two samples (emAT and mcAT parameters per grid). The correlation coefficient r can take values between -1 and +1 that specify the strength and the sign of the direction of the relationship. If r = 0, no linear relationship between the variables is evident.

Correlations with r ≥ 0.5 are considered as high, and r ≥ 0.7 as very high (Cohen, 1988).

### Receiver operating characteristic - ROC curve

The ROC curve originated from the signal detection theory and is used in medicine as a tool for the evaluation of known diagnostic tests. Transmitted to the two methods of attention measurement the ROC curve or AUC (area under the curve) measures the goodness of this measure (mcAT parameters) to predict the occurrence or absence of the variable of the other method (emAT parameters). There are four possibilities of prediction: right positive, false positive, right negative and false negative.

The ROC curve is created by a diagram of the *correct positive rate* (known as the *“hit ratio” or “sensitivity”*) and is deducted from the *false positive rate* (also known as *“one minus specificity”*) (Fig. 2), wherein the threshold of the classifier (the AT parameter) is continuously varied. The closer to the diagonal, the more the right-positive rate corresponds to false positive rate – which is expected of the right-positive frequency of a random process equivalent. Thus, the greater the area under the curve (AUC), the better is the prediction; and thus, the agreement between the two variables (64, 65).

*Sensitivity* i.e. probability of detection (see 2 refs. above) – measures the *number* of positives that are correctly identified as such (e.g. the percentage of mouse clicks that resemble *true eye fixations*). *Specificity* (also called the true negative rate) measures the number of negatives that are correctly identified as such (e.g., the *number* of mouse clicks not resembling eye fixations, false alarms). Thus, *sensitivity* quantifies the avoiding of false negatives, as specificity does for false positives. For any test, there is usually a trade-off between these measures. This trade-off can be represented graphically as a receiver operating characteristic, *ROC curve* (Fig. 2).

A perfect predictor would be described as 100% sensitive and 100% specific; but any predictor will possess a minimum error bound (Bayes error rate). The ROC curve is the *sensitivity as a function of fall-out*, i.e. the proportion of non-relevant measures that are retrieved, out of all non-relevant measures available: In general, if the probability distributions for both detection and false alarm are known, the ROC curve can be generated by plotting the cumulative distribution function, i.e. the area under the probability distribution for the discrimination threshold of the detection probability in the y-axis versus the cumulative distribution function of the false-alarm probability in x-axis.

In the medical field, the divisions to assess the test accuracy are: An AUC value ≥ 0.7 is considered acceptable, ≥ 0.8 good acceptable, and ≥ 0.9 as *excellent* (65).

Another method used is the assessment of the predictive power of a computer model as a reference value, where the inter-subject variance or inter-subject homogeneity to validate emAT is employed. (53, 66). For this purpose, the AUC for the prediction of the emAT data for one half of the subjects is determined by the other half of the subjects. The higher the value, the lower the variance – or the higher the homogeneity among emAT subjects. This value is considered to be the theoretically achievable AUC or the upper limit of a computer model for predicting fixations (63, 66).

To address the *first hypothesis*, i.e. to determine the relationship between the two samples, the correlation coefficient (correlation of measurement of pairs per grid) and the AUC are calculated based on the total stimulus material. A high positive correlation between mcAT and emAT values is observed for both parameters – *contact, attention* – if AUC is significantly above the chance level of 0.5.

To determine the ROC curve using the present emAT and mcAT data it was necessary to clarify which contact or attention value of an emAT sample was interpreted as “seen”; as only the “not seen” and “seen” classes were used for the “seen” calculation.

Therefore, *three possible limits* were initially set per parameter and the curves for all three were calculated and compared.

As no reference values were found in the literature for this problem, the limits were set primarily by theoretical considerations on the respective central test limits (at contact: 0.3; in attention: 0.05) and set for calculation of the AUC values of the individual categories used. As the measured values were not normally distributed and thus a prerequisite for calculating the significance tests for correlations was not met (67), the confidence intervals of the correlation coefficients were determined using bootstrapping (68). Bootstrapping is a resampling technique used to obtain estimates of summary statistics. It can refer to any test or metric that relies on random sampling with replacement. Bootstrapping allows assigning measures of accuracy to sample estimates. It allows estimation of the sampling distribution of almost any statistic using random sampling methods.

### Fixation/Click rates, scatter plots and difference histograms

**Figure 3.**
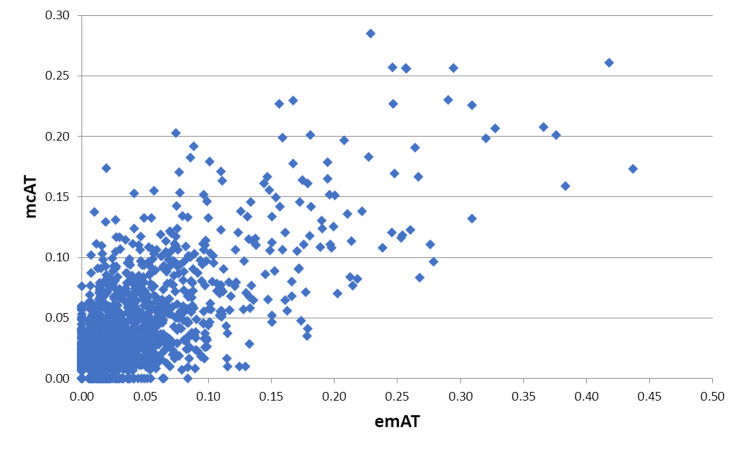
. Attention values mcAT as a function of emAT for all pictures.

Figure 3 shows the attention values of mcAT as a function of emAT for all pictures. Graphically, it demonstrates a close relationship between the two methods

## Results

To address the second hypothesis, the two summary measures were calculated and compared for each category of images. In the main “Simple” category in particular, higher compliance – i.e. a higher correlation coefficient and a higher AUC – between mcAT and emAT was postulated than in the adjacent category “complex”, as well as in the “human/animal” category compared to the other categories of the same level. In order to check whether the detected correlation coefficients of the various categories differ significantly from each other, the online calculator used significance testing with correlations suggested by Wolfgang Lenhard & Alexandra Lenhard (69), specifically the test for comparison of two correlation coefficients of independent samples. The average attention value (n = 10) in the emAT test amounted to between 0.00 and 0.44. This means that one single grid received up to 44% of all fixations while an image was being viewed. The highest number of attention value in the mcAT test was 0.28, i.e. one single grid received up to 28% of all clicks. With respect to the contact values, in both experiments all values were between 0, i.e. fields that nobody paid any attention to, and 1, i.e. fields all subjects did notice. The distribution of emAT and mcAT value pairs per grid-box is graphically depicted in Figure 3 by means of a scatter plot.

### First Research Question - The ROC curve results

In nearly all image categories the correlation amounted to r = 0.76 (attention) and r = 0.71 (contact). Both correlations are highly significant and greater than zero (P <;0.001). The confidence intervals determined by bootstrapping (.72 to 0.78 (attention) and 0.68 to 0.74 (contact)), also indicate that, in our sample, the correlation coefficients can be classified as high or very high.

**Figure 4.**
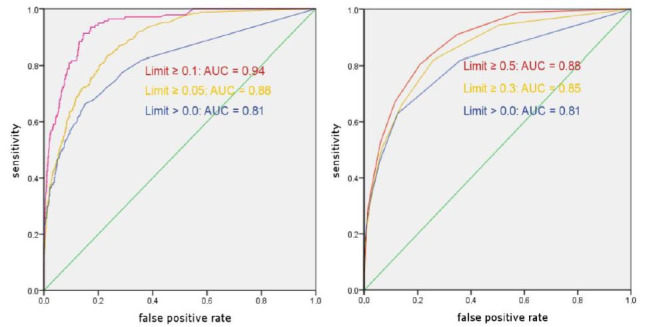
ROC curves with different limits. Left: attention values; right: contact values.

Figure 4 shows the ROC curves for the entire stimulus material with three different thresholds. In the attention value for the selected limit of 0.05, the AUC size is 0.88 with a confidence interval of 0.87 to 0.90. In the contact value with the limit 0.3, the AUC size is 0.85 with a confidence interval of 0.84 to 0.87. Both values are thus significantly different from 0.5 (p <;0.001).

**Table 1.**
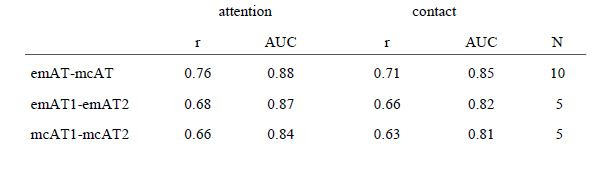
Correlation coefficients of the between-subjects (1st row) compared to the within-subject correlations (2nd and 3rd rows).

The above described correlation coefficient and the AUC for the subject’s internal prediction of fixations is 0.88 (attention) and 0.85 (contact); the correlation within the ET-sample amounts to r = 0.68 (attention) and r = 0.66 (contact). This means there is a closer link between the mcAT and emAT data (n = 10) than between the emAT data of the one with the other sub-group (n = 5) (see Table 1).

Inter-subject variance of the emAT data was determined through a within-subject analysis. Correlation coefficients and AUC values were lower than the results from the between-subject design.

The finding that mouse clicks were more similar to eye fixations than eye fixations to themselves seems hard to understand at first glance. It may be a consequence of the way we generate eye movements. Eye movements are very fast in three respects: We perform many movements per second, eye movements are generated with a short response latency, and saccades are the fastest movements we can generate. This may lead to the effect that eye movements are somewhat inexact, more often than Mouse clicks. We can observe the inaccuracy of eye movements in any eye-tracking recording: Fixations on a given target are located in an area around the target that is about one degree of visual angle. In comparison, Mouse clicks seem to have a higher accuracy than eye fixations.

Statistically speaking, the inaccuracy of eye movements leads to noise in the recorded data. When we compare eye fixation data with eye fixation data, we compare two noisy sources. In contrast, when we compare gaze data with click data, we compare one more and one less noisy source. This explains, why the comparison of two eye fixation data yields a higher difference than the comparison of eye fixation data with click data.

### Second Research Question – the picture categories and emAT vs. mcAT

**Figure 5.**
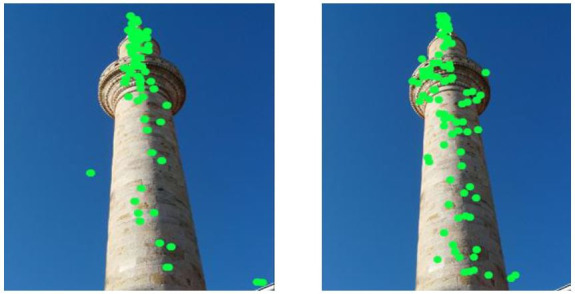
Comparison of the viewing (left) and click data (right) image InAS #5 (inanimate, artificial, simple)

The correlation coefficients of the individual images demonstrated a large scattering width depending upon the different picture categories (see Fig.1 for reference). The picture with the highest obtained correlation (r = 0.95 (*attention*) and r = 0.94 (*contact*)) was within the category inanimate artificial simple (InAS) (Fig.5).

**Figure 6.**
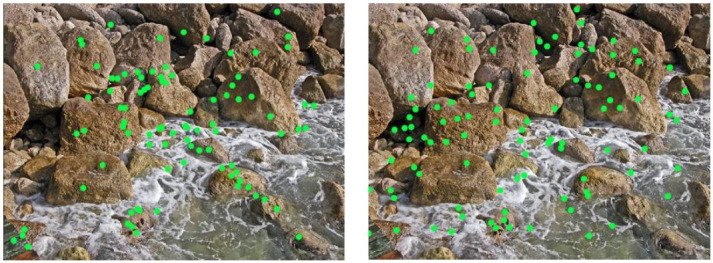
Comparison of the viewing (left) and click data (right) image InNC #8 (inanimate, natural, complex)

The picture for both parameters with the lowest correlation (r = 0.22 (*attention*) and r = 0.17 (*contact*)) was one of eight images from the category *inanimate natural complex* (InNC, Fig. 6).

Of the total of 64 images obtained, seven (*attention*) and four (*contact*) images showed a correlation of only r <;0.5, i.e. a low effect size. On the other hand, six (*attention*) and two images (*contact*) showed a correlation of r > 0.9.

**Figure 7.**
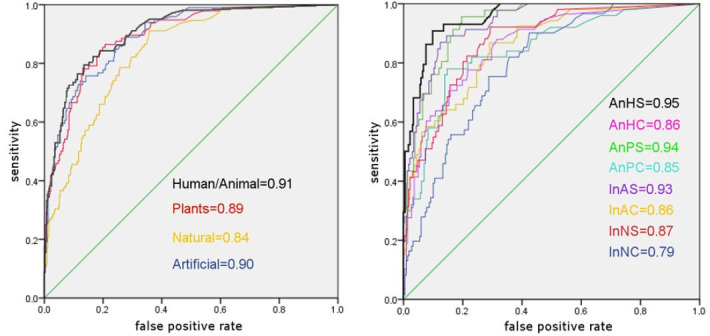
Comparison of the AUC values: Left: Basic categories, attention; right: Super categories, 2nd level, attention

Similar results were obtained in the evaluation of the AUC of the individual super categories represented in Figure 7.

All AUC values differ significantly from chance level with an AUC of 0.5, and all categories are at least in an acceptable, almost good range (65).

But in this statistical analytical method there are also large, significant differences between the super categories. The image category with the highest values was AHS (*animate human simple*) with an AUC of 0.95 (*attention*) or 0.94 (*contact*).

With an AUC of 0.79, INC (*inanimate natural complex*) is the category with the lowest correlation between emAT and mcAT data, and *attention* values are medium (0.5). For *contact*, this applies to the category inanimate artificial complex (IAC) with an AUC of 0.771, similar to the AUC of category INC (0.774).

Generally, attention showed slightly higher values than contact. The “human/animal” category consistently showed higher values than neighboring categories. Most of the test results regarding significance between the different categories were similar for both attention and contact. Both the attention and contact values clearly show the difference between the correlations for the AHS (animated human simple) category and almost all other categories. Both also very clearly differ regarding the “easy” and “complex” image categories. Furthermore, in the “human/animal” super category, the differences with respect to all three categories on the same level were highly significant. The “human/animal” super category also shows a highly significant difference between the “animate” and “inanimate” categories.

We conclude with the observation that the comparable significant similarity of this data demonstrates convincingly the close link and resemblance of the mcAT and emAT methods for searching, recognizing and perceiving the images shown.

## Discussion

We investigated the conformity of the mcAT-measurement data (clicks) (n = 10) and the emAT measurement data (fixations) (n = 10). This was based on 64 photographs that were viewed by our subjects. These images were divided into eight categories of our classification scheme. The comparison of click and fixation rates demonstrated that clicks yielded highly similar results to eye movements within our paradigm. In accordance with suggestions in the literature, clicks were on average slightly slower, and occurred with smaller numbers than fixations.

To what extent fixations and clicks match each other, and whether there are differences depending upon the stimulus material?

We found a highly positive and significant correlation between mcAT and emAT data. The AUC values were significantly (p<;0.01) above chance level of 0.5 with correlations of r = 0.76 for attention values, and r = 0.71 for contact values. Inter-subject variance of the emAT data was determined through a within-subject analysis. Correlation coefficients as well as AUC-values were below results from the between-subject design. Due to the small number of participants (5 vs. 5 of the within-subject designs compared to 10 vs. 10 of between-subject designs) the variance was high.

This was comparable to other studies with a higher number of subjects: Rajashekar, van der Linde, Bovik and Cormack (70) reported a larger inter-subject variance with r = 0.75, compared to r = 0.68 (attention) and 0.66 (contact). Their study was based on a sample of emAT with n = 29, and on stimuli excluding images with top-down features.

Interestingly, using a calibration function built by an algorithm (71) it was possible to predict where a user will click with a mouse: The accuracy of the prediction was about 75% ‒ which points to a high correlation as also shown here.

Our second hypothesis was also confirmed: The correlation coefficient and AUC values of individual categories differed from each other. Both the basic eight categories as well as the super-categories showed significant differences for many variations between categories of the same level. As expected, we found that the main “simple” category showed significantly higher correlation values (r = 0.81) for attention when compared to the “complex” category (r = 0.66).

The same was true for the “human/animal” super-category (r = 0.82) with respect to the other three categories of the second level “plants” (r = 0.74), “natural” (r=0.74) and “artificial” (r=0.72). This is important evidence for the validity of the mcAT procedure. It has been demonstrated by many researchers that viewers prefer images of humans and animals. The preference for pictures of humans and animals, especially of faces that are simply structured, is also reflected in the eight fundamental categories. These show by far the highest correlations of 0.86 for attention and an excellent AUC of 0.95. Thus, this category is significantly superior to all others. The lowest correlation of mcAT and emAT data is demonstrated by images that represent complex natural structures, but still with a high linear correlation. As print ads and websites often depict people with clearly defined AOIs such as title, motif, slogan, and brand logo, we conclude that the mcAT procedure is highly suitable for measuring attention in this type of stimuli.

It is interesting to note that our results are in line with most previously published reports on our categories: Animate (human/animal plants), Inanimate *(artificial natural*), as we demonstrate in the following descriptions.

The face recognition system is capable of extremely fine within-category judgments to recognize and discriminate between faces and different facial expressions displayed by the same face (72, 73). To support this ability, it has been proposed that a separate system evolved to mediate face recognition.

These results indicated the existence of an experience-independent ability for face processing as well as an apparent sensitive period during which a broad but flexible face prototype develops into a concrete one for efficient processing of familiar faces. (74).

A cortical area selective for visual processing of the human body was described by Downing, Jiang, Shuman and Kanwisher (75). Despite extensive evidence for regions of human visual cortex that respond selectively to faces, few studies have considered the cortical representation of the appearance of the rest of the human body. They presented a series of functional magnetic resonance imaging (fMRI) studies revealing substantial evidence for a distinct cortical region in humans that responds selectively to images of the human body, as compared with a wide range of control stimuli. This region was found in the lateral occipitotemporal cortex in all subjects tested (n = 19) and apparently reflected a specialized neural system for the visual perception of the human body.

Several lines of evidence suggest that animate entities have a privileged processing status over inanimate objects – in other words, that animates have priority over inanimates. The animate/inanimate distinction parallels the distinction between “living” and “nonliving” things that has been postulated to account for selective deficits in patients (for a review, see Capitani, Laiacona, Mahon, & Caramazza, (76)). Animates belong to the general category of living things.

Their studies revealed better recall for words denoting animate than inanimate items, which was also true with the use of pictures. The findings provided further evidence for the functionalist view of memory championed by Nairne and co-workers (77, 78).

Evidence from neuropsychology suggests that the distinction between animate and inanimate kinds is fundamental to human cognition. Previous neuroimaging studies have reported that viewing animate objects activates ventrolateral visual brain regions, whereas inanimate objects activate ventromedial regions.

### Comparison of eye and hand movements from a neuro-bioengineering perspective

A saccade made to a target that appears eccentric to the point of fixation is sometimes called a ‘reflexive’ (or ‘stimulus-elicited’) saccade in contrast to those made in situations that depend more heavily upon voluntary (or ‘endogenous’) cognitive control processes (for example when directed by a simple instruction “look to the left”). Most saccades are essentially voluntary in nature, as an observer can always decide not to move the eyes. Also, if the time and place of a target’s appearance can be predicted, an anticipatory saccade often occurs before the target itself appears, or too briefly subsequently for visual guidance to have occurred.

When reaching for targets presented in peripheral vision, the eyes generally begin moving before the hand (79, 80, 81, 82 ,83, 84) This is the case because much of the delay in hand movement onset, relative to eye movement onset, can be attributed to the greater inertia of the arm. Recent studies demonstrate that the motor commands underlying coordinated eye and hand movements appear to be issued in close temporal proximity and that commands for hand movement may even precede those for eye movement (80, 85, 86).

**Figure 8.**
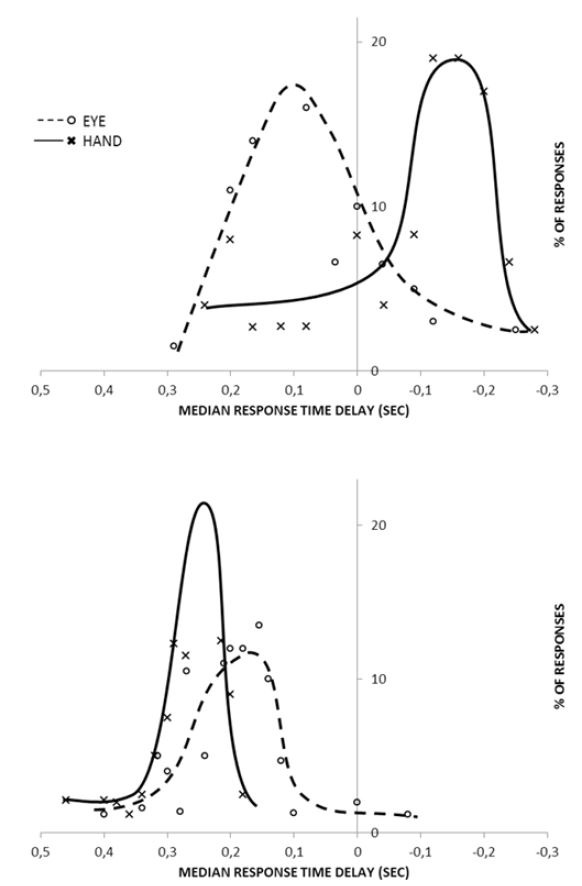
The dependence of median response time (i.e. latency) to frequency of repetitive square-wave patterns (Stark, 1968). Histograms of response time delays for hand and eye. Top:predictive square waves at 1.2 cps. Bottom: random target.

Furthermore, hand movement can influence saccadic initiation. Saccadic reaction time (SRT) is greater when eye movement is accompanied by hand movement compared to when the eyes move alone (87, 88), and SRT and hand reaction time (HRT) both increase when reaching for targets in contralateral versus ipsilateral space (89). In addition, in eye-hand coordination saccades are faster when accompanied by a coordinated arm movement (90). Because of the large variation in response characteristics of both, hand and eye systems with different subjects, both systems have been compared by measuring the systems´ responses simultaneously (49, 88). When eye and hand responses were recorded simultaneously to random steps and predictive regular steps, the eye shows shorter response times than those of the hand (Fig.8).

The eye muscles have considerable power with respect to their constant load, the eyeball, and show faster rise times than the hand, especially when tracking rapidly alternating signals. With random targets, the hand response lags behind the eye response due to the eye’s considerably smaller load. At moderate frequencies of 0.7 to 1.0 cps the hand develops prediction faster and to a greater extent than the eye. At higher frequencies of >1.1 cps the hand shows considerable prediction, while the median eye response time starts to lag despite the higher frequency characteristics of the actual movement dynamics of the eye. In general, at low frequencies there is some correlation evident between eye and hand response.

Obviously, eye movement is not necessary for hand movement. Conversely, the physical movement of the hand appears to help the eye movement system: Adequate eye tracking may occur with comparatively high frequencies, if the hand is tracking and may not occur if the hand is still. When hand tracking improved eye performance and then stopped, the eye performance deteriorated significantly (49, 88).

With respect to our paradigm, this means that the mcAT method might help the sequence of eye fixations that must go along with the mouse clicks when viewing and perceiving the test images. This is particularly true in test settings where sensory-motor actions tend to be time-optimal due to limited time: This was the case in our test set with the time limit of 5 sec.

Interestingly, Bednarik, Gowases, & Tukiainen (91) showed that users with gaze-augmented interaction outperformed two other groups – using mouse or dwell time interaction – on several problem-solving measures: they committed fewer errors, were more immersed, and had a better user experience. As mentioned, the slower manual action of the hand/finger-movement when activating the mouse may well be due to the physiological properties rather than solely on to the hidden cognitive processes of augmented gaze in problem solving as the authors speculate. A combined eye and mouse interaction may show an even more successful result, since hand and eye movements in coordination could act faster and more precisely in many situations (88).

In continuation of the early results by Stark (49), Helsen, Elliott, Starkes, and Ricker (92)studied eye and hand movement coordination in goal directed movements. They found a remarkable temporal relationship between the arrival of the eye and the hand on the target position. Because point of gaze always arrives on the target area first, at roughly 50% of the hand response time, there is ample opportunity for the eye to provide extra-retinal information on target position either through oculomotor efference or visual and proprioceptive feedback resulting from the large primary saccade. They demonstrated an invariant relationship between the spatial-temporal characteristics of eye movements and hand kinematics in goal-directed movement that is optimal for the pickup of visual feedback. Interestingly, the natural coupling between eye and hand movements remains intact even when hand movements are merely imagined as opposed to being physically executed. So, it appears that the bioengineering and neurophysiological literature shows indeed a solid background for the highly correlated relationship of eye and mouse movements firstly described by Egner and Scheier in 2002.

Bieg, Chuang, Fleming, Reiterer and Bülthoff (93) showed, when target location was unknown (quasi random), the eyes lead the mouse by 300 ms on average. When the approximate location of the target was known (i.e. predictive), the cursor often led gaze in acquiring the target, and fixations on the target occurred later in the pointing process. This again corresponds to the early results of Stark and Navas inasmuch the degree of prediction of a target influences the result.

Knowledge about the target location is likely to be very important especially in non-laboratory settings. This was shown by Liebling and Dumais (94) presented an in-situ study of gaze and mouse coordination as participants went about their normal activities before and after clicks. They analyzed the coordination between gaze and mouse, showing that gaze often leads the mouse, about two thirds of the time, and that this depends on type of target and familiarity with the application; but not as much as previously reported, and in ways that depend on the type of target.

Rodden, Fu, Aula and Spiro (95) tracked subjects´ eye and mouse movements and described three different types of eye-mouse coordination patterns. However, they found that the users were not easy to classify: each one seemed to exhibit several patterns of the three types to varying degrees. There was also substantial variation between users in high-level measures: The mean distance between eye and mouse ranged from 144 to 456 pixels, and the proportion of mouse data points at which the eye and mouse were in the same region ranged from 25.8% to 59.9%. This corresponds with Huang, White and Dumais (96) results. During Web search, Huang et al. found that eye and mouse most often were correlated. The average distance between the eye and mouse was 178 px, with the differences in the x-direction being much larger (50 px) than in the y-direction (7 px).

Later, Chen et al. (46) also reported that in web browsing during certain subtasks, mouse and gaze movements were very often correlated. They found that the average distance between mouse and gaze was 90 pixels during transitions from one area of interest (AOI) to another, and that 40% of the distances were closer than 35 pixels.

In summary, as gaze provides a measure of attention, knowing when the mouse and gaze are aligned i.e. highly correlated, this confirms the usability of the mouse as indicator of attention as shown by Egner and Scheier (62).

### Limitations of emAT and mcAT 

Both methods used in this study, emAT and mcAT, were geared towards determining the respondents’ current attention location. However, as attention is an internal process in the brain, we are measuring external responses (gaze direction, mouse location) and use the measured data to infer the actual attention location. These external responses as well as their measurements are subject to noise (Tab.2) (Appendix 2).

**Table 2.**
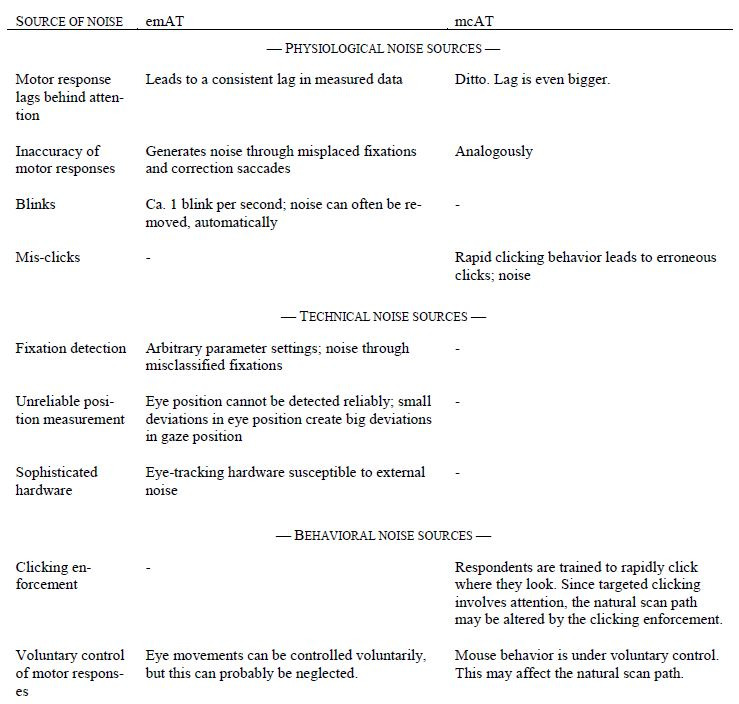
Comparison between the two attention measurements used in the present article. See also Appendix 3

Also, past research has found a correlation between gaze and cursor positions (46, 95, 96, 97 ,98) and that cursor movements can be useful for determining relevant parts of the Web page with varying degrees of success (96, 99, 100, 101). Cursor interaction spans a variety of behaviors including reading, hesitating, highlighting, marking, and actions such as scrolling and clicking. Huang et al. (96) showed that user, time, and search task (to a lesser extent) each contribute to the variation in gaze-cursor alignment. The gaze and cursor positions are also better aligned when the gaze position is compared to a future cursor position. Furthermore, by distinguishing between five different cursor behaviors—inactive, examining, reading, action, and click—one might get a better idea of the strength of alignment. Identifying these behaviors was beyond the scope of this paper. For further discussion of these problems see Huang et al. (96). Demšar and Çöltekin (102) investigated the actual connection between the eye and the mouse when searching web pages. They found that there seemed to be natural coupling when eyes were not under conscious control, but that this coupling breaks down when instructed to move them intentionally. Therefore, they suggested that for natural tracing tasks, mouse tracking could potentially provide similar information as eye-tracking and so be used as a proxy for attention. Since our paradigm used a clear-cut task that asked our subjects to consciously coordinate eye and mouse movements, this aspect of Demšar and Çöltekin appears not to be relevant in our context.

### Starting points for further evaluations and investigations

Based on the results and limitations of this study, and the explanations of the theoretical part of this work, recommendations for further research can be derived that could complete the results of this work and lead to a broader assessment of the contribution and validity of mcAT. Firstly, it describes aspects that are already present in the data that can be evaluated in secondary analyzes. A further method would be *the analysis of spatial correspondence of fixations and clicks*, which would provide an extension to the viewing or clicking motion paths. The question of whether clicking may be influenced by short-term memory in relation to the presented stimuli cannot be answered here in the context of the evaluated tests but should be studied. The evaluation of the qualitative survey could also shed some light on possible gaze and click strategies with mcAT. Furthermore, the influence of the number and size of the grids on the images varies and should be checked systematically to determine whether the effects found in this study can be confirmed.

Another possibility would be to select from the present stimulus material only those images that are semantically oriented instead of using grids. AOIs could be defined in a next step and evaluation methods for these new AOIs then repeated. For checking the coincidence of time of fixation and clicks, other parameters such as the viewing time and time to contact for both could be calculated and compared. Another question is how precise are spatial mouse clicks set? The variation of the presentation duration for certain looking/click strategies of the subjects would enable a time-based evaluation in which the extent to which the match of gaze and click data is also dependent upon the presentation duration could be checked.

## Conclusion

The aim of this work was a fundamental review of the suitability of mcAT to measure the attention of participants by registering clicks on the computer screen. The validation was carried out under a between-subject design based on eye tracking (emAT), the standard method of attention measurement. The focus of the investigation was the analysis of the spatial relationship of the measured data of both methods. With respect to findings reported in the literature, it was assumed that, in general, a close relationship between gaze and click data exists. However, the extent of the relationship varies for different image categories. Both hypotheses were confirmed. As eye tracking (emAT) is predominantly accepted as the valid method for measuring attention, we can conclude that mouse-click tracking (mcAT) is similarly highly valid.

A further finding of our research was that this innovative method obtains particularly valid results with stimuli that are simply structured and where humans or animals are shown. This suggests that the use of mcAT for attention measurement is well suited for print ads, and thus for advertising research a valid alternative to eye tracking, with benefits regarding practicability. Due to some emAT’s restrictions, we suggest that, in other fields, mcAT could replace the registration of eye movements in cases where eye tracking may be inaccurate or technically unfeasible and provides a promising additional method for usability research (103).

## Ethics and Conflict of Interest

The author(s) declare(s) that the contents of the article are in agreement with the ethics described in http://biblio.unibe.ch/portale/elibrary/BOP/jemr/ethics.html and that there is no conflict of interest regarding the publication of this paper.

## Appendix

### Appendix 1

Utilized stimulus material

Animate

human/animal simple complex plants simple complex

Inanimate

artificial simple complex natural simple complex

### Appendix 2

Instructions for mcAT trial

Follow your viewing of the image with the computer mouse, so that the fixation points are transferred into mouse clicks. In order to optimize your eye-hand coordination, get a feel for the necessary click speed; initially you will have to complete a short click training. The first step is done when you have completed the training successfully. Now the real test starts, in which you have to follow your viewing process for 5 seconds for 64 images, each preceded and followed by a separating fixation cross. Throughout the trial period try to keep your click speed constant with a minimum frequency of at least 1-2 clicks per second. Note that it is not possible to stop the click test in between. Also, try to focus and concentrate fully until to the end of the test (duration: 7.5 minutes). At the end of the click test there will follow a brief survey of the pictures that you have just viewed. Tell us if you have seen the following 16 images mentioned in the test or not: 16 images (1 per page; 8 true, 8 wrong; 1 from each category). This will allow us to evaluate the technical quality of your click performance.

### Appendix 3

Sources of noise

Sources of noise in classical eye-tracking (emAT):

- Gaze direction is not always identical to attention direction (Anderson et al., 2015; Chun & Wolfe, 2005). It can be assumed that, under normal viewing conditions, the gaze is preceded by attention. This introduced a delay in our measured data.
- When the gaze follows the attention direction, it typically performs saccades that target the current attention direction. However, saccades are not always precise. When a saccade misses the actual attention direction, an additional saccade (correction saccade) is performed. In such a case, emAT measures multiple saccades and fixations while only one attentional shift has been performed. The additional saccades and fixations are not corresponding to attention; they are noise. Of course, saccades are possible that represent a strategic under-shoot or over-shoot.
- Blinks are an additional source of noise. However, we attempted to remove all blinks from the emAT data in this study.
- An underlying assumption of emAT is that fixations directly correspond to attention directions. However, it is often hard to classify data from the eye-tracker into fixations. Depending upon the choice of parameters of the fixation detection algorithm (temporal, spatial), it classifies differing portions of the trajectory as fixation. There is no optimal parameter regime; therefore, there will always be some mis-detected fixations, which can be seen as noise.
- The measurement of gaze direction is technically demanding, because small differences in the eye position correspond to large differences in the gaze position. Therefore, the measurement has to have a high accuracy on the raw data level to avoid big mistakes on the level of gaze positions. Head movements in all directions create additional difficulty. Altogether, the technical difficulties lead to more or less noisy data.

Analogously to the physiological and technical sources of noise in emAT, mcAT must deal with both kinds of noise sources:

- The hand is much slower than the eye. It can, therefore, be assumed that the lag between the attention location and the measured position is even bigger than in emAT.
- Mouse movements are also not precise.

*Stimulus material:* There remains a certain arbitrariness in the derivation and definition of image categories. In addition, in reality mixed forms of categories such as simultaneous displays of plants and animals usually occur.

*Sample*: Although both samples are similar in their demographic structure, subjects consisted exclusively of students aged between 18 and 26 years. This accounts for a small population segment only. One of the advantages of the mcAT procedure is that it allows for a large population and many different audiences to perform the mcAT.

*Statistical analysis:* As determination of semantically-oriented AOIs involves certain disadvantages in terms of the stimulus material used, the division of the images in grids was the better alternative. However, the optimal number of fields per frame could not be determined. As each emAT and mcAT data pair per AOI counted as one case in the analysis, a large or too large field or case number might mean small, essentially insignificant effects get rated as significant. For this reason, we calculated and assessed effects across both linked measures (correlation and AUC) and parameters (attention and contact). To calculate the significance between correlation values of the individual picture category, the online calculator (ref: http://www.socscistatistics.com/tests/pearson/ ) for comparison of two correlation coefficients was used. The emAT and mcAT metrics are independent in terms of subjects and methods of measurement, but they are not independent with respect to the stimulus material: i.e. the stimuli are the same for both maneuvers, but different in time and dynamics.
